# Evaluating Treatment Modalities for Reducing Recurrence in Central Giant Cell Granuloma: A Narrative Review

**DOI:** 10.3390/dj12090295

**Published:** 2024-09-19

**Authors:** Flamur Aliu, Donika Bajrami Shabani, Iliriana Aliu, Etleva Droboniku Qeli, Gerta Kaçani, Luca Fiorillo, Aida Meto

**Affiliations:** 1 Private Dental Clinic “I Dent”, 10000 Prishtina, Kosovo; flamuraliu.oms9@gmail.com (F.A.); ailiriana@gmail.com (I.A.); 2 Department of Dental Pathology and Endodontics, Faculty of Medicine, University of Prishtina, 10000 Prishtina, Kosovo; 3 Department of Dental Therapy, Faculty of Dental Medicine, University of Medicine, 1005 Tirana, Albania; etleva.droboniku@umed.edu.al; 4 Department of Prosthetics, Faculty of Dental Medicine, University of Medicine, 1005 Tirana, Albania; gerta.kacani@umed.edu.al; 5 Department of Dentistry, Faculty of Dental Sciences, University of Aldent, 1007 Tirana, Albania; lfiorillo@unime.it; 6 Department of Biomedical and Dental Sciences, Morphological and Functional Images, University of Messina, 98100 Messina, Italy; 7 Multidisciplinary Department of Medical-Surgical and Odontostomatological Specialties, University of Campania “Luigi Vanvitelli”, 80121 Naples, Italy; 8 Department of Public Health Dentistry, Dr. D.Y. Patil Dental College and Hospital, Dr. D.Y. Patil Vidyapeeth, Pimpri, Pune 411018, Maharashtra, India; 9 Laboratory of Microbiology and Virology, Department of Surgery, Medicine, Dentistry and Morphological Sciences with Interest in Transplant, Oncology and Regenerative Medicine, University of Modena and Reggio Emilia, 41125 Modena, Italy; 10 Department of Conservative Dentistry and Endodontics, Dr. D.Y. Patil Dental College and Hospital, Dr. D.Y. Patil Vidyapeeth, Pimpri, Pune 411018, Maharashtra, India

**Keywords:** central giant cell granuloma, recurrence, treatment modalities, surgical excision, curettage, corticosteroid injection

## Abstract

Treating central giant cell granuloma (CGCG) is challenging due to high recurrence rates and variable therapy responses. This study examines the efficacy of various treatments in reducing CGCG recurrence. A literature review explored outcomes of surgical excision, curettage, intralesional corticosteroid injection, and adjuvant therapy, considering factors like lesion location, size, and histological features. Aggressive surgical techniques such as en bloc resection were found to potentially lower recurrence rates compared to conservative approaches. However, treatment should be tailored to individual patient needs. Further research is needed to confirm these findings and improve treatment strategies. A concise literature review was conducted using PubMed, MEDLINE, and Google Scholar, focusing on papers published from 1986 to 2024. Search terms included “central giant cell granuloma”, “recurrence”, “treatment modalities”, and “surgical excision”. Studies reporting recurrence rates and treatment outcomes for CGCG were analyzed. Twenty-nine studies were reviewed, including six studies on surgical excision and curettage, eight studies on intralesional corticosteroid injections, six studies on calcitonin therapy, five studies on interferon-alpha therapy, and four studies on the therapy with denosumab. Analysis indicated that aggressive surgical treatments like en bloc resection were associated with lower recurrence rates compared to conservative methods. Predictors of recurrence included lesion size (>3 cm), location (mandible), and aggressive histopathological features. Aggressive surgical excision combined with nonsurgical methods may lower recurrence rates, while conservative techniques remain viable in some cases. Further prospective research is needed to validate these findings and enhance CGCG treatment options.

## 1. Introduction

Central giant cell granuloma (CGCG) is a rare but significant entity, accounting for roughly 10% of benign jawbone lesions primarily affecting individuals in their twenties to forties [1]. It is characterized by the proliferation of fibrous tissue within the bone, which features areas of hemorrhage, clusters of multinucleated giant cells, and occasional formations of immature bone trabeculae [2]. This way, CGCG presents as a locally aggressive lesion in the jawbones [3].

Initially described as “giant cell reparative granuloma”, this condition is notable for its potential to recur if not appropriately managed. Proper treatment is essential to reduce the risk of recurrence [4]. The condition exhibits a higher prevalence in females, with a reported female-to-male ratio of 2:1 [1]. Although CGCG predominantly occurs in the mandible, particularly in the anterior region, body, and ramus, it can also be present in the maxilla, albeit less frequently. Notable anatomical structures, such as the maxillary sinus, can be involved when CGCG occurs in this region [5]. This involvement can lead to clinical challenges, including sinus obstruction, displacement of teeth, and potential impacts on nasal cavity structures. While CGCG in the maxilla is not as common as in the mandible, its occurrence warrants careful attention due to the complex anatomical considerations. The presence of CGCG in the maxilla highlights the necessity for precise imaging and customized surgical strategies to manage these cases effectively [5]. 

Approximately 50% of CGCG lesions are located anterior to the first molar in the alveolus, with around 35% extending across the midline. The rarity of multiple CGCG lesions is noted, with occurrences sometimes associated with systemic conditions like Noonan syndrome and neurofibromatosis type 1 [5]. While most cases are solitary, the presence of multifocal or bilateral lesions may indicate underlying conditions such as hyperparathyroidism or cherubism, necessitating thorough investigation [6].

The etiology of CGCG remains multifaceted and continues to be debated within the medical community. Early hypotheses suggested an inflammatory, reactive, or possibly endocrine basis, while more contemporary views lean towards a neoplastic origin [7]. Fibroblasts are considered the main proliferative cells, secreting cytokines that attract monocytes and encourage their differentiation into multinucleated giant cells [7]. Immunohistochemical analyses have confirmed that these multinucleated giant cells within CGCG lesions are indeed osteoclasts, offering a further understanding of their pathogenesis [7]. Moreover, immunohistochemistry has identified these large cells as osteoclasts, as evidenced by the presence of positive markers such as CD68+, TRAP+, V-ATPase+, Rank+, and carbonic anhydrase II+ [8].

Imaging studies play a crucial role in the diagnosis and characterization of CGCG. Typical findings include loculated, expansile lesions with wavy septations, moderate enhancement, and occasional evidence of cortical perforation and tooth root resorption [8]. On computed tomography (CT), these lesions often present as midline expansile masses in the mandible, exhibiting multiloculated features with septations perpendicular to the cortex. Larger lesions may demonstrate scalloping and cortical dehiscence, underscoring their potential for local bone destruction [8]. 

Despite its benign classification, CGCG poses a therapeutic challenge due to its propensity for recurrence. Treatment strategies vary and may include surgical excision, curettage, intralesional corticosteroid injections, and adjuvant therapies. However, the optimal approach to reduce recurrence rates remains unclear, highlighting the need for further research and clinical trials [9,10]. Chuong et al. categorized CGCG into non-aggressive and aggressive forms based on their biological behavior and radiographic characteristics, guiding treatment decisions accordingly [11]. While non-aggressive lesions are more common, recognizing and appropriately managing aggressive variants is crucial for achieving favorable treatment outcomes. Although most lesions are non-aggressive, aggressive forms have also been identified [11,12,13].

Having a high recurrence rate after therapy, CGCG is a therapeutic challenge for clinicians. Several therapeutic options have been used, including surgical excision, curettage, intralesional corticosteroid injection, and adjuvant therapy. However, the best way to lower recurrence rates is unclear. This study aims to examine the efficacy of various treatment modalities in reducing CGCG recurrence and identifying characteristics that influence treatment outcomes.

## 2. Materials and Methods

A comprehensive narrative review was performed using PubMed, MEDLINE, and Google Scholar to identify relevant studies published between 1986 and 2024. The search focused on key terms such as “central giant cell granuloma”, “recurrence”, “treatment modalities”, and “surgical excision”. This review consolidates existing literature to evaluate the effectiveness of various treatment approaches for CGCG and identify factors influencing recurrence rates, aiming to provide a comprehensive summary of current knowledge while highlighting emerging trends and gaps in the research. The variability in case report quality, follow-up protocols, and reporting standards was acknowledged, which limited the possibility of conducting a comprehensive quantitative analysis.

## 3. Research Background

Jaffe first described the lesion in 1953 as an intraosseous lesion characterized by a fibrous stroma with high cellularity, multiple foci of hemorrhage, aggregated multinucleated giant cells, and irregular trabeculae of woven bone [1]. Since Jaffe’s initial description, many authors have proposed different perspectives regarding the lesion’s origin, biological behavior, and treatment protocols, often resulting in either under- or overestimation. Despite these varying viewpoints, the lesion’s benign nature and its high recurrence rate have been consistently recognized up to the present time.

In 1986, Chuong et al. conducted a pivotal clinicopathologic study highlighting the importance of distinguishing between aggressive and non-aggressive lesions due to the higher recurrence rate associated with aggressive lesions. They emphasized that the term “central giant cell lesion” (CGCL) should be used to differentiate between the histological characteristics and biological behavior of these two types [11]. This distinction was crucial for determining appropriate treatment strategies. Following this, Auclair et al. conducted a comprehensive comparative analysis of the clinical, histologic, and histomorphologic features of CGCG in both long bones and jaws. Their study suggested that these entities represent a spectrum of the same disease, with variations primarily based on the patient’s age and the lesion’s site of occurrence [12]. This perspective provided a broader understanding of CGCG, emphasizing the need for tailored treatment approaches based on specific clinical and biological parameters.

Treatment regimens for CGCG should be guided by the classification of the lesion as either aggressive or non-aggressive [10]. Over the past decades, various treatment options have been explored, including curettage, intralesional corticosteroid injections, subcutaneous injections of calcitonin, and surgical excision. Despite these diverse approaches, no single effective treatment strategy for CGCG has been universally established. The choice of treatment often depends on the lesion’s characteristics and the patient’s specific condition, underscoring the need for individualized treatment plans.

The curettage of CGCLs has been a mainstay treatment of choice for many years [14]. Studies indicate that the recurrence rates for CGCG vary from 11% to 49% [15]. However, when curettage is performed alone, the recurrence rate for aggressive lesions increases significantly, ranging from 30% to 70% [14].

The risk of recurrence and the potential complications of large lesions, such as tooth loss, damage to the inferior alveolar nerve, and harm to the sinuses and nose, have prompted the development of various non-surgical and adjunctive treatments [16]. The lack of a single effective treatment is due to the diverse biological behavior of CGCG [11]. In cases refractory to medical treatment and surgical curettage, surgical resection may be considered [17]. Although surgical procedures can be effective, they come with disadvantages such as cosmetic impact and functional disturbances [18]. A recent case report highlighted the successful treatment of a large, destructive CGCG through enucleation, aggressive curettage, and peripheral ostectomy while preserving mandibular continuity [19]. However, the literature indicates that this approach is associated with moderate to high recurrence rates.

Another study recommends using conservative surgery as the initial management strategy for CGCG to avoid the high morbidity associated with more radical surgical approaches [20]. This study identified factors such as multilocularity, the surgical method used (curettage only), the lesion’s location, and the bigger size (>5 cm), as significant contributors to the high recurrence rates [7,20]. These findings underscore the importance of individualized treatment plans and the ongoing need for research to optimize therapeutic strategies for CGCG.

The first nonsurgical treatment proposed for CGCG was intralesional corticosteroid injections, which have shown varied success [16,21,22,23]. Corticosteroids are used in managing CGCG based on their mechanisms of blocking bone resorption and inducing osteoclast apoptosis [23]. In 1994, Terry and Jacoway first applied an injection of a 50/50 mixture of triamcinolone (Kenalog) and local anesthetic directly into the lesion once a week for six weeks, recommending 2 cc of solution for every 1 cc of visible lesion on orthopantomography [22].

The literature on the efficacy of corticosteroids has shown mixed results. Approximately 50% of cases report a favorable response, characterized by complete resolution or hypercalcification of smaller lesions [21]. Corticosteroids have been found to be particularly effective in smaller, unilocular, and well-circumscribed lesions, as they can readily penetrate all parts of the lesion without missing any areas [22]. This success contrasts with their effectiveness in multilocular lesions, where achieving comprehensive treatment coverage can be more challenging. These findings underscore the importance of lesion size and morphology in determining the suitability and potential success of corticosteroid treatment for CGCG.

Other authors have also reported successful outcomes from using intralesional corticosteroid injections, particularly for solitary lesions and in children at risk of losing their teeth or tooth germs [24]. The recurrence rate appears to correlate with the initial size of the lesion before treatment [17]. While corticosteroids can be highly effective in treating smaller lesions, they have consistent disadvantages, including extended application times, issues with patient compliance, and potential systemic side effects [25].

In 2012, da Silva et al. reported a case where bisphosphonates were combined with intralesional corticosteroid injections. This approach resulted in minimal radiolucency around the root apices of the involved teeth two years post-treatment, and after four years, new areas of bone formation were observed on panoramic radiography with no signs of recurrence [26]. A long-term follow-up study in 2019 further supported the effectiveness of this combined treatment, especially in children with CGCG [27].

It should be noted that patients treated successfully with intralesional corticosteroid injections might still require minor surgical interventions to achieve desired esthetic outcomes. Additionally, the follow-up period after intralesional corticosteroid injections has been reported to range from 10 months to 8 years, which is necessary to monitor recurrence and evaluate therapeutic outcomes [1].

In the management of CGCG, calcitonin therapy has emerged as a promising non-invasive treatment option. Calcitonin can be administered via subcutaneous injections or intranasal spray, providing flexibility in treatment, particularly beneficial for pediatric patients [28].

In 1993, Harris first used subcutaneous calcitonin for one year to treat aggressive CGCG cases [29]. Pogrel later extended the administration period to approximately 18 months, suggesting that calcitonin’s effectiveness might be due to its inhibition of osteoclastogenesis via calcitonin receptors on some of the giant cells in these lesions [24]. The binding of calcitonin to these cells may inhibit osteoclast formation, thereby preventing bone destruction [1].

Subcutaneous administration is commonly used, with dosages adjusted based on the lesion’s size and aggressiveness. However, the intranasal route offers a less invasive alternative, particularly advantageous for children to minimize discomfort and ensure better compliance. This distinction is crucial when considering potential side effects and the patient’s ability to tolerate the treatment over extended periods, which can last up to 24 months depending on the response to therapy [28].

Despite these advantages, nasal delivery is usually avoided today due to the variation in absorption [15]. Current research focuses on developing powder formulations for nasal delivery of salmon calcitonin, incorporating various absorption enhancers and stabilizers to improve its bioavailability [30,31]. However, the study by de Mendonça et al. presents a long-term follow-up of a CGCG case treated with intralesional corticosteroid injections and bisphosphonates [27]. Intralesional corticosteroids reduce inflammation and inhibit osteoclast activity, minimizing bone resorption and lesion progression, while bisphosphonates inhibit osteoclast-mediated bone resorption, making them effective in managing CGCG [27].

Radiographic evidence of resolution following calcitonin therapy typically becomes apparent after 6 to 9 months, with treatment potentially needing to continue for up to 24 months [17]. Calcitonin administration has been considered an alternative to surgery, particularly for aggressive lesions and young patients. However, the lengthy duration of therapy poses a significant disadvantage due to decreased serum calcium levels and an increased risk of peptic ulcers [25,32].

A randomized double-blinded placebo-controlled study evaluating the efficacy of calcitonin in managing CGCG found no significant difference between calcitonin and placebo [33]. Despite this, various reviews have reported favorable results with calcitonin therapy, with some even noting total remission [24,25]. Some authors recommend administering 100 IU of subcutaneous calcitonin injections for larger, ill-defined, and non-aggressive lesions that threaten vital structures, as well as for small aggressive lesions with slow progression [34]. These mixed results highlight the need for further research to fully understand the potential and limitations of calcitonin therapy for CGCG.

The rationale behind alpha-interferon therapy lies in its ability to suppress growth factors that drive the angiogenic component of lesions [14,35]. Introduced by Kaban et al. in 1999, this therapy demonstrated efficacy in treating aggressive giant lesions following unsuccessful resections [14]. Post-enucleation, alpha-interferon is administered subcutaneously at a dose of 3 million units/m^2^ per day. Treatment duration typically spans 6 to 8 months, with monitoring through urine fibroblast growth factor measurements to mitigate recurrence risk [24].

Alpha-interferon effectively halts the rapid growth of aggressive lesions and can even reduce their size, although surgical intervention may still be necessary for complete resolution. Despite its efficacy, the therapy is associated with side effects such as drug-induced lupus erythematosus, pancreatitis, fever, fatigue, and flu-like symptoms [14,24,25,35]. Nonetheless, it offers a less radical approach and has been shown to significantly lower recurrence rates.

Denosumab, a human monoclonal antibody targeting receptor activator of nuclear factor-κB ligand (RANKL), is FDA-approved for treating osteoporosis in postmenopausal women and shows promise in managing these lesions [36,37]. By inhibiting osteoclast activity, particularly in RANKL-positive giant cell lesions, denosumab offers targeted molecular-level therapy [36,37]. Treatment involves subcutaneous administration of 120 mg monthly, adjusted to 60 or 70 mg for patients under 45–50 kg, supplemented with vitamin D and calcium, typically over 12 months [38].

The radiological response can be observed after approximately 5.5 doses, leading to ossification in all reported cases and lesion size reduction in three instances [39]. Indications for initiating therapy with denosumab include large lesions and those refractory to other treatment modalities [38]. A recent cohort study identified lesion size (>5 cm), aggressive lesion type, and fewer than 12 initial doses of denosumab as factors contributing to recurrence after therapy [39]. Given the potential risk of recurrence with denosumab, further long-term follow-up studies are needed.

## 4. Results

The review systematically analyzed 29 studies that investigated the efficacy of various treatment modalities for CGCG. These included 6 studies on surgical excision and curettage, 8 studies on intralesional corticosteroid injections, 6 studies on calcitonin therapy, 5 studies on alpha-interferon therapy, and 4 studies on denosumab therapy.

Comparative analysis revealed that aggressive surgical approaches, such as en bloc resection, consistently exhibited lower recurrence rates when compared to more conservative methods like curettage and corticosteroid injections, particularly when combined with adjunctive therapies. Notably, lesion characteristics such as size (especially larger lesions), location (predominantly in the mandible), and histopathological features (such as multilocularity) were identified as significant predictors of recurrence across various treatment modalities.

However, a significant limitation of the reviewed studies was the paucity of long-term follow-up data, particularly for newer and emerging treatment options like denosumab. Despite promising initial results, the durability of response and long-term outcomes remain uncertain. Table 1 summarizes the comparative analysis of these treatment modalities for CGCG, highlighting their respective impacts on recurrence rates and the need for further research to establish optimal management strategies.

The review extracted data from various studies on treatment modalities for CGCG, encompassing surgical excision, curettage, intralesional corticosteroid injections, and adjunctive therapies. Factors critical to the comparative analysis included lesion size, location, and histopathological characteristics. Surgical excision, particularly aggressive approaches like en bloc resection, consistently demonstrated lower recurrence rates compared to more conservative methods such as curettage and corticosteroid injections. Lesion size (often larger lesions), mandibular location, and histopathological features like multilocularity emerged as significant predictors of recurrence across different treatment approaches.

## 5. Discussion

Central giant cell lesions have often caused confusion among clinicians due to the interchangeable use of terminologies and overlapping histopathological characteristics with other jaw pathologies. Historically, these lesions have been termed as regenerative entities. However, with the accumulation of more data over the years, it is now appropriate for clinicians to refer to them as CGCGs or CGCLs. It is also important to note that the aggressive subtype of CGCG should not be referred to as a “giant cell tumor”. While both conditions involve giant cells and share histological features such as the presence of multinucleated giant cells, current literature indicates that they are distinct entities with different clinical behaviors, pathological characteristics, preferred locations, and management strategies. Therefore, using precise terminology is crucial to avoid confusion and to ensure clarity and accuracy in diagnosis, treatment planning, and scholarly communication.

The classification of CGCG into aggressive and non-aggressive forms, as proposed by Chuong et al. (1986) remains a foundational guideline for the clinical management of these lesions [11]. In their seminal clinicopathologic study, Chuong et al. identified distinct differences in the biological behavior of CGCG, with aggressive lesions exhibiting higher recurrence rates and more invasive growth patterns. This classification is critical when determining the appropriate treatment approach, as aggressive lesions often require more extensive surgical interventions, such as en bloc resection, to minimize the risk of recurrence. In contrast, non-aggressive CGCG can often be managed with more conservative methods, such as curettage or intralesional corticosteroid injections. The findings of Chuong et al. have been consistently validated in subsequent studies, emphasizing the importance of tailoring treatment strategies based on lesion behavior to optimize patient outcomes. In our review, we observed similar trends, with aggressive CGCG exhibiting higher recurrence rates, particularly in larger lesions and those located in the mandible, aligning with Chuong’s classification.

Various treatment modalities have been explored, aiming to minimize recurrence and improve long-term outcomes. Surgical approaches, including curettage and en bloc resection, have traditionally been employed, often yielding lower recurrence rates compared to non-surgical methods [23]. However, the choice of surgical technique can significantly influence outcomes, with aggressive surgical resection demonstrating better efficacy in reducing recurrence, particularly for larger and more aggressive lesions [16].

Intralesional corticosteroid injections have also been widely used, primarily targeting smaller, well-circumscribed lesions [35]. Corticosteroids act by reducing inflammation and inhibiting osteoclastic activity, thereby promoting lesion consolidation and reducing the likelihood of recurrence [32]. Despite their effectiveness in certain cases, their utility in larger, multilocular lesions remains contentious due to challenges in achieving comprehensive treatment coverage [20].

Calcitonin therapy has been investigated as an adjunctive treatment for CGCG, aiming to inhibit osteoclastogenesis and thereby reduce bone resorption within the lesion [21]. Studies have reported variable outcomes, with some demonstrating significant radiographic resolution and reduced recurrence rates in selected patient groups. However, the prolonged treatment duration and potential side effects, such as decreased serum calcium levels, limit its widespread application [23,24].

Alpha-interferon therapy has shown promise in managing aggressive CGCG lesions refractory to conventional treatments [13,34]. By suppressing angiogenic factors crucial for lesion growth, alpha-interferon can effectively halt disease progression and reduce lesion size. Despite its efficacy, the therapy is associated with notable side effects, necessitating careful patient selection and monitoring [13,34].

Denosumab, a monoclonal antibody targeting RANKL, has emerged as a novel therapeutic option for CGCG, particularly in cases resistant to conventional therapies [36]. By inhibiting osteoclast activity, denosumab promotes ossification within the lesion and reduces its size, offering a targeted approach to managing this challenging condition [37,38]. However, concerns regarding its long-term safety profile and potential for rebound effects post-treatment warrant further investigation.

The comparative analysis of these treatment modalities reveals significant variability in their efficacy and safety profiles, influenced by lesion characteristics such as size, location, and histopathological features. Larger lesions and those exhibiting aggressive behavior often necessitate more aggressive treatment approaches to achieve satisfactory outcomes [23,24]. The heterogeneity in treatment responses underscores the importance of individualized treatment planning based on comprehensive clinical assessment and histopathological evaluation.

Despite advancements in therapeutic options, several limitations persist in the current literature. Long-term follow-up data are often sparse, particularly for newer modalities like denosumab, which hinders the assessment of durability and recurrence rates over extended periods. Furthermore, the variability in study designs and patient cohorts complicates direct comparisons between different treatment strategies, emphasizing the need for standardized protocols and multicenter collaborative studies to establish evidence-based guidelines.

In conclusion, optimizing the management of CGCG requires a nuanced understanding of its biological behavior and treatment response. While surgical excision remains a cornerstone of therapy, especially for aggressive lesions, the integration of adjunctive therapies such as corticosteroids, calcitonin, alpha-interferon, and denosumab offers promising avenues for improving treatment outcomes. 

## 6. Future Research Directions

The management of CGCG continues to evolve, driven by ongoing research aimed at enhancing treatment efficacy and minimizing recurrence rates. Several promising avenues warrant exploration to address current knowledge gaps and optimize clinical outcomes. These include the following:

*Long-term Efficacy of Novel Therapies:* Evaluate the long-term efficacy and safety profiles of emerging therapies such as denosumab and alpha-interferon. Comprehensive studies with extended follow-up periods are essential to assess treatment durability and identify any potential late effects or recurrence patterns post-therapy.
*Standardization of Treatment Protocols:* Establish standardized treatment protocols for CGCG management, considering lesion size, location, and histopathological features. Comparative studies should focus on defining optimal treatment algorithms to guide clinicians in selecting the most appropriate therapeutic approach based on individual patient characteristics.
*Biomarker Identification:* Investigate potential biomarkers associated with CGCG recurrence and treatment response. Biomarker discovery could facilitate early detection of disease progression, predict treatment outcomes, and aid in monitoring therapeutic efficacy.
*Alternative Therapeutic Targets:* Explore novel therapeutic targets beyond conventional approaches. Research into molecular pathways involved in CGCG pathogenesis could uncover new therapeutic avenues, including targeted molecular therapies or immunomodulatory agents.
*Patient-reported Outcomes:* Incorporate patient-reported outcomes and quality-of-life measures into clinical trials. Assessing patient perspectives on treatment efficacy, functional outcomes, and psychosocial impact would provide valuable insights into the holistic management of CGCG.
*Pediatric Considerations:* Address specific considerations in pediatric patients, including growth implications, skeletal development, and long-term sequelae of treatment. Tailored approaches that account for age-related factors and developmental stages are crucial for optimizing outcomes in this vulnerable population.
*Cost-effectiveness Analysis:* Conduct cost-effectiveness analyses to evaluate the economic implications of different treatment strategies. Comparative assessments should weigh upfront treatment costs against long-term benefits, including recurrence rates and quality-adjusted life years.

By addressing these research priorities, the field can advance towards personalized, evidence-based care for CGCG, improving therapeutic outcomes and enhancing patient well-being.

## 7. Conclusions

Optimizing the management of CGCG requires a nuanced understanding of both the biological behavior of the lesions and patient-specific factors. While aggressive surgical excision has traditionally been the cornerstone of treatment, particularly for aggressive and large lesions, there is a growing emphasis on less invasive options. Recent trends have favored medical management approaches, including intralesional corticosteroid injections and adjunctive therapies like calcitonin and alpha-interferon, especially for cases where surgical intervention may lead to significant morbidity, such as in pediatric patients or those with extensive lesions. The shift towards these conservative treatments aims to reduce the potential for substantial tissue or tooth loss and other complications associated with more radical surgical methods. Our current management protocol aligns with this trend, prioritizing non-surgical methods where feasible and reserving surgical interventions for cases that do not respond to medical therapies or present with life-threatening symptoms. This tailored approach is supported by the recognition that CGCG treatment should be individualized, taking into account the lesion’s size, location, and patient health status. Future research should continue to explore the efficacy of these treatments, with a particular focus on long-term outcomes and quality of life. Therefore, we strongly recommend the consistent use of the terms central giant cell granulomas (CGCGs) or central giant cell lesions (CGCLs) to ensure clarity and accuracy in diagnosis, treatment planning, and scholarly communication.

## Figures and Tables

**Table 1:** Treatment modalities for reducing recurrence rate in CGCG.

Treatment Modality	Efficacy	Recurrence Rates	Side Effects	References
Surgical Curettage	High	Moderate to High	Tissue loss, structural damage	[19,20]
Steroid Injections	Moderate	Low	Pain, swelling at the injection site, immunosuppression	[1,27]
Calcitonin Therapy	Variable	Variable	Low serum calcium levels, peptic ulcers	[24,25]
Denosumab Therapy	High	Low	May have hypocalcemia, osteonecrosis of the jaw	[38,39]

## Data Availability

The data presented in this study are available on request from the corresponding authors.
